# Epidemiological and genomic analyses identify drug classes associated with Lewy body dementia

**DOI:** 10.1002/alz.088858

**Published:** 2025-01-09

**Authors:** Sonja W Scholz

**Affiliations:** ^1^ National Institute of Neurological Disorders and Stroke, Bethesda, MD USA

## Abstract

**Background:**

Lewy body dementia (LBD) is the second most common neurodegenerative dementia after Alzheimer’s disease which remains poorly understood. Disease‐modifying therapies for LBD are critically needed. We performed a population‐based case‐control study of U.S. Medicare participants with LBD and matched controls to identify drugs that are significantly associated with disease risk.

**Method:**

A total of 148,170 U.S. Medicare and 1,253,043 controls were included in the study. We estimated the association of LBD risk with 1,017 prescription drugs. We then applied genetic correlation analyses to genomic data generated for LBD and cardiovascular traits to investigate causal effects.

**Results:**

Using broadly adjusted logistic regression models, we discovered significantly reduced LBD risk associated with drugs used to treat cardiovascular diseases (antihypertensives: odds ratio = 0.72, 95% confidence interval = 0.70–0.74, p‐value = 0; cholesterol‐lowering agents: odds ratio = 0.85, 95% confidence interval = 0.83–0.87, p‐value = 0; and antidiabetics medications: odd ratio = 0.83, 95% confidence interval = 0.62–0.72, p‐value = 0). Notably, antidiabetic medications were associated with a larger risk reduction among Black LBD patients than other racial groups. Genetic correlation analyses identified shared genetic risk between LBD and low‐density lipoprotein cholesterol levels, hypertension, and type 2 diabetes mellitus.

**Conclusions:**

Our data suggest that the genetic architecture of LBD and cardiovascular traits overlap and that assiduous management of cardiovascular risk factors may be beneficial in this this patient population.

**Reference**: Scholz SW et al., Brain Commun. 2023 Dec 18;6(1):fcad346.

